# Supplementary material for: Systemic Inflammation and the Increased Risk of Inflamm-Aging and Age-Associated Diseases in People Living With HIV on Long Term Suppressive Antiretroviral Therapy
Source: Front Immunol. 2019 Aug 27;10:1965. doi: 10.3389/fimmu.2019.01965 (PMC6718454; doi:10.3389/fimmu.2019.01965)
Supplement: Supplementary file 1 [file Table_1.DOCX]

**Supplementary Table 1.** Proteins with significant association with Telomere length after adjustment for HIV status, age and gender in ART and HIVNC

| Protein | Estimated difference | 95% CI | P-value |
| --- | --- | --- | --- |
| CXCL1 | 0.3921 | (0.002, 0.782) | 0.0486 |
| MMP-10 | 1.7882 | (0.936, 0.640) | 0.0001 |
| CD40 | 0.7412 | (0.029, 1.454) | 0.0417 |
| CX3CL1 | 1.4148 | (0.200, 2.630) | 0.0232 |
| OSM | -0.7886 | (-1.403, -0.174) | 0.0127 |
