# Supplementary material for: Systemic Inflammation and the Increased Risk of Inflamm-Aging and Age-Associated Diseases in People Living With HIV on Long Term Suppressive Antiretroviral Therapy
Source: Front Immunol. 2019 Aug 27;10:1965. doi: 10.3389/fimmu.2019.01965 (PMC6718454; doi:10.3389/fimmu.2019.01965)
Supplement: Supplementary file 2 [file Image_1.pdf]

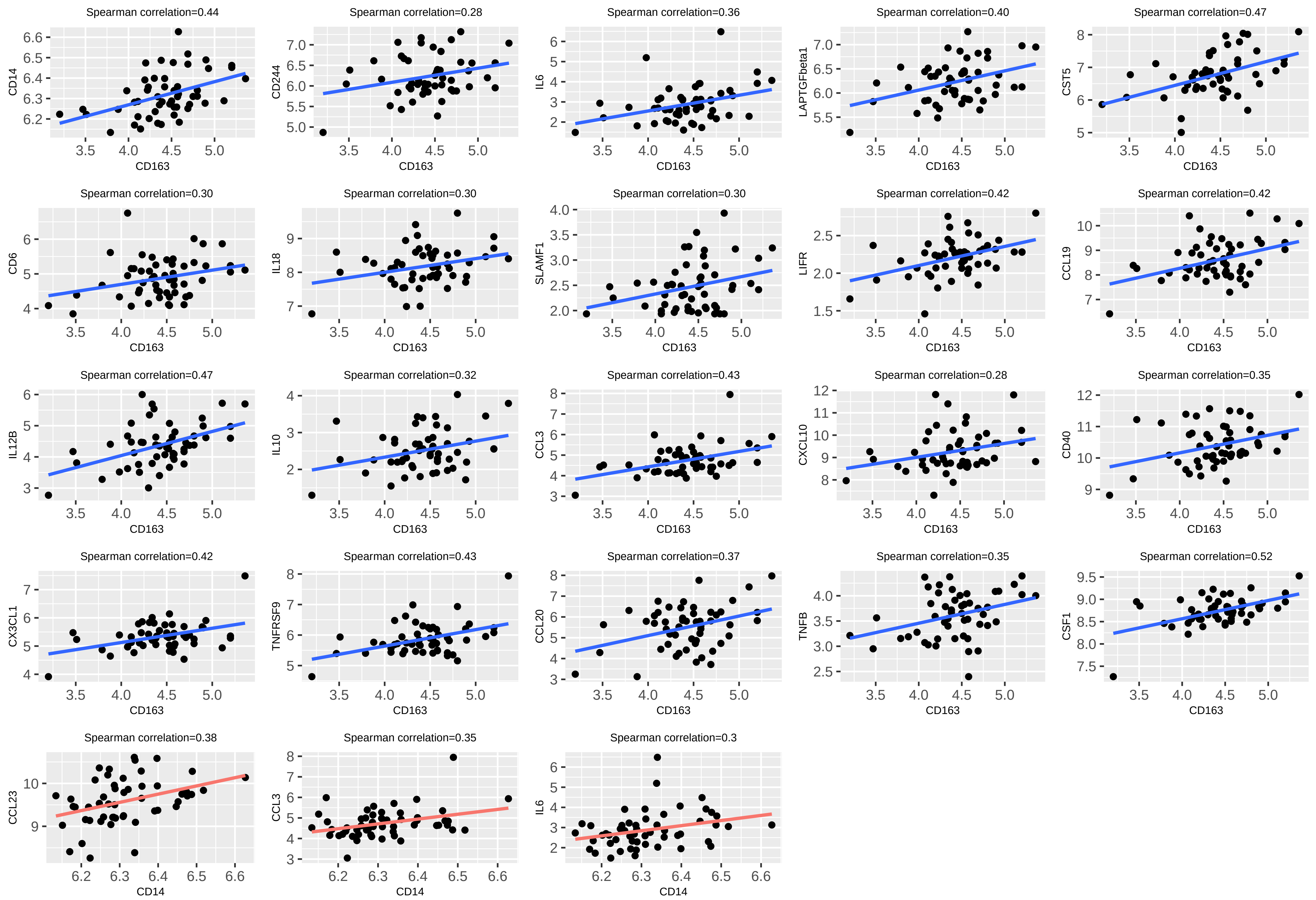

Supplementary Figure 1: Correlation Scatter plots of CD163 and CD14 with significant spearman correlation(p<0.05)
